# Two new species of dictyostelid cellular slime molds in high-elevation habitats on the Qinghai-Tibet Plateau, China

**DOI:** 10.1038/s41598-018-37896-7

**Published:** 2019-01-09

**Authors:** Pu Liu, Yue Zou, Shu Li, Steven L. Stephenson, Qi Wang, Yu Li

**Affiliations:** 1 Engineering Research Center of Chinese Ministry of Education for Edible and Medicinal Fungi, Jilin Agricultural University, Changchun, 130118 P. R. China; 2 Department of Biological Sciences, University of Arkansas, Fayetteville, AR 72701 USA

## Abstract

Dictyostelid cellular slime molds (dictyostelids) are key components of soil microbes. The Qinghai-Tibet Plateau is characterized by unique and important forest types because of the considerable range in elevation which exists. During the period of 2012, 2013 and 2016, 12 species of dictyostelids were yielded from samples collected in this region, including two new species and three new records for China. Six other species were new records for this region. Ontogeny, morphology, ultrastructure and systematic molecular analyses (SSU & ITS) of *D. minimum* and *D. multiforme* confirm that they are Goup 4 new species. The ornamentation of the surface of dictyostelids’ spores is the first time to be observed until now. In the SSU phylogenetic tree generated in the present study, *Synstelium*, not assigned to order and family before, was assigned to the clade Acytosteliaceae in the Acytosteliales firstly. To our knowledge, the study reported herein is the first investigation of dictyostelid biodiversity carried out at elevations above 2000 m. Sorocarp size, sorus size, spore length, ratio of sorus and sorophore, and ratio of sorus and spore size were positively correlated with increasing elevation and no linear correlated with forest type, according to the results of linear regression analysis.

## Introduction

Dictyostelids, the second largest group of slime molds, have both animal-like (protozoan) and fungus-like characteristics. The vegetative phase consists of single-celled amoeboid forms that live in the soil, where they feed upon bacteria and other microbes, grow, and multiply until the available food supply is exhausted. When this happens, the amoeboid forms aggregate together in large numbers to form multi-celled pseudoplasmodia, which then give rise to fruiting bodies (sorocarps) that consist of supportive stalks and unwalled sori containing propagative spores^1,2^.

Although the first dictyostelid was described by Brefeld in 1869^3^, relatively little work was done on the Dictyosteliaceae until 1941, when Harper, Arndt and Raper began their studies of these organisms^2^. The first ecological survey was carried out in the forests of southern Wisconsin in the United States, when Cavender and Raper^4^ sampled six sites located along a moisture gradient. Their samples were processed with the use of a quantitative method of isolation^5^. This experiment showed that dictyostelids are affected by environmental factors, especially moisture^6^. It is now known that there are a number of factors which can affect the distribution and abundance of dictyostelids, including the physiographic regime sampled^7,8^, soil pH, soil type, climatic conditions, forest type^6,9,10^, elevation^7,10–12^, and latitude^13^. Some species of dictyostelids have been grouped into abundance categories, including very common, common, rare, and very rare^9,14–16^.

Environmental factors such as elevation and pH appear to have a predominant effect on patterns of biodiversity in dictyostelids, while the effects of forest management are secondary^7^. Species biodiversity is generally very low under dry conditions, although Romeralo *et al*.^17^ found *Polysphondylium violaceum* Bref. to be prominent in drier vegetation types. In the Iberian Peninsula, dictyostelids abundance was reported to be highest in colder and wetter environments, which suggests that this group favors relatively cold places with high levels of water availability^6^.

Dictyostelids have evolved a number of different structures in response to the different environments in which they occur. Some species (e.g., *Dictyostelium septentrionalis* [Cavender 1978], now *D. septentrionale*) form a thick sorophore to help keep this structure erect as an adaptation for fruiting in cool temperatures; other species (e.g., *D. rhizopodium* Raper & Fennell, now *Hagiwaraea rhizopodium* [Raper & Fennell] S.Baldauf, S.Sheikh & Thulin) form root-like basal crampons in order to help the sorocarp remain erect longer in tropical habitats; and members of the genus *Acytostelium* produce small sorocarps, which is probably an adaptation associated with their narrow niche requirements^17^.

According to the traditional morphology-based classification, dictyostelids were placed in the class Dictyosteliomycetes under the phylum Protozoa. This class was considered to include one order, two families and four genera^18^ that are distinguished morphologically by differences in sorophore composition and branching pattern. However, data from a phylogenetic analysis based on 18S ribosomal RNA (18S rRNA) and α-tubulin, indicated that none of these three genera are monophyletic, with the dictyostelids divided into four groups^19^ and eight groups^20^, respectively. However, Sheikh *et al*.^21^ proposed a new classification based on unique 18S rRNA sequence signatures. These data provided a new insight into the taxonomy of the dictyostelids, and as a result of this new classification, two families, 9 genera and 92 new combinations were recognized at the level of species and variety.

The first records of dictyostelids from China were reported by Ronglin Bai^22^, who listed five species, including four species of *Dictyostelium* and one species of *Polysphondylium*, isolated from samples of soil and fallen leaves of broadleaf forests collected in Beijing, Jilin Province, and Shanxi Province, China. In the same year, Yeh and Chien^23^ reported the occurrence of *D. brefeldianum* H.Hagiw., *D. giganteum* B.N. Singh, *P. violaceum* and *P. pallidum* Olive (now *Heterostelium pallidum* [Olive] S.Baldauf, S.Sheikh & Thulin) in samples collected from nine localities located in broadleaf deciduous forests in Taiwan, China. In addition, several new records and new species of dictyostelids have since been reported from a number of localities in China, including Taiwan, Jilin, and Tibet, Heilongjiang, Jiangsu, Inner Mongolia, Hainan, Shanxi and Hubei provinces^23–41^.

The Qinghai-Tibet Plateau, the highest plateau in the world, is located in the west of China between 73.19–104.47°E and 26.00–39.47°N. This region has long been known as the roof of the world. Most of the plateau is characterized by relatively high elevations, often in excess of 4000 m, but some portions reach only about 2000 m. The range of different elevations leads to a number of unique vegetation types. Studies of dictyostelids in this region are exceedingly limited and consist of a single previous report of a species new to science^35^.

The primary objective of the study reported herein was to increase our knowledge of dictyostelid biodiversity and abundance at high elevations (>2000 m) and in ecologically complex habitats. Samples for isolation of these organisms were collected from nine sites on the Qinghai-Tibet Plateau. The dictyostelids recovered from these samples were studied, both for their morphological features and also with molecular markers. The species biodiversity of dictyostelids on the Qinghai-Tibet Plateau is discussed in relation to previous studies and concepts relating to the overall ecology of these organisms.

## Results

**Figure 1:**
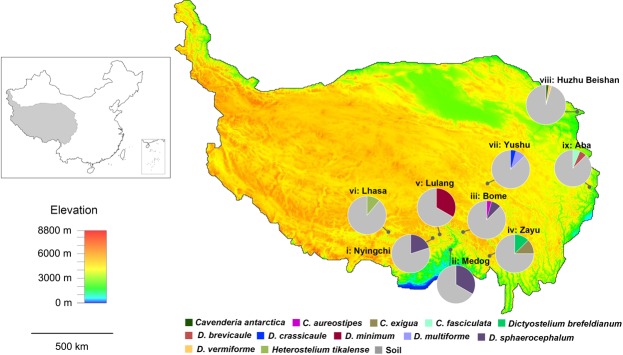
Map showing the collecting sites in China and the nine collecting sites on the Qinghai-Tibet Plateau. Information on the species of dictyostelids recovered and the proportion of the total each species represented is provided for each of the nine sites. The different color from red to blue of elevation scale represented different elevations from 8800 m to 0 m. (i) the Sejila Mountain National Forest Park, Nyingchi; (ii) Medog; (iii) Bome; (iv) Zayu; (v) Lulang; (vi) Lhasa; (vii) Yushu; (viii) Huzhu Beishan National Forest Park; (ix) the Jiuzhaigou National Forest Park, Aba.

**Figure 2:**
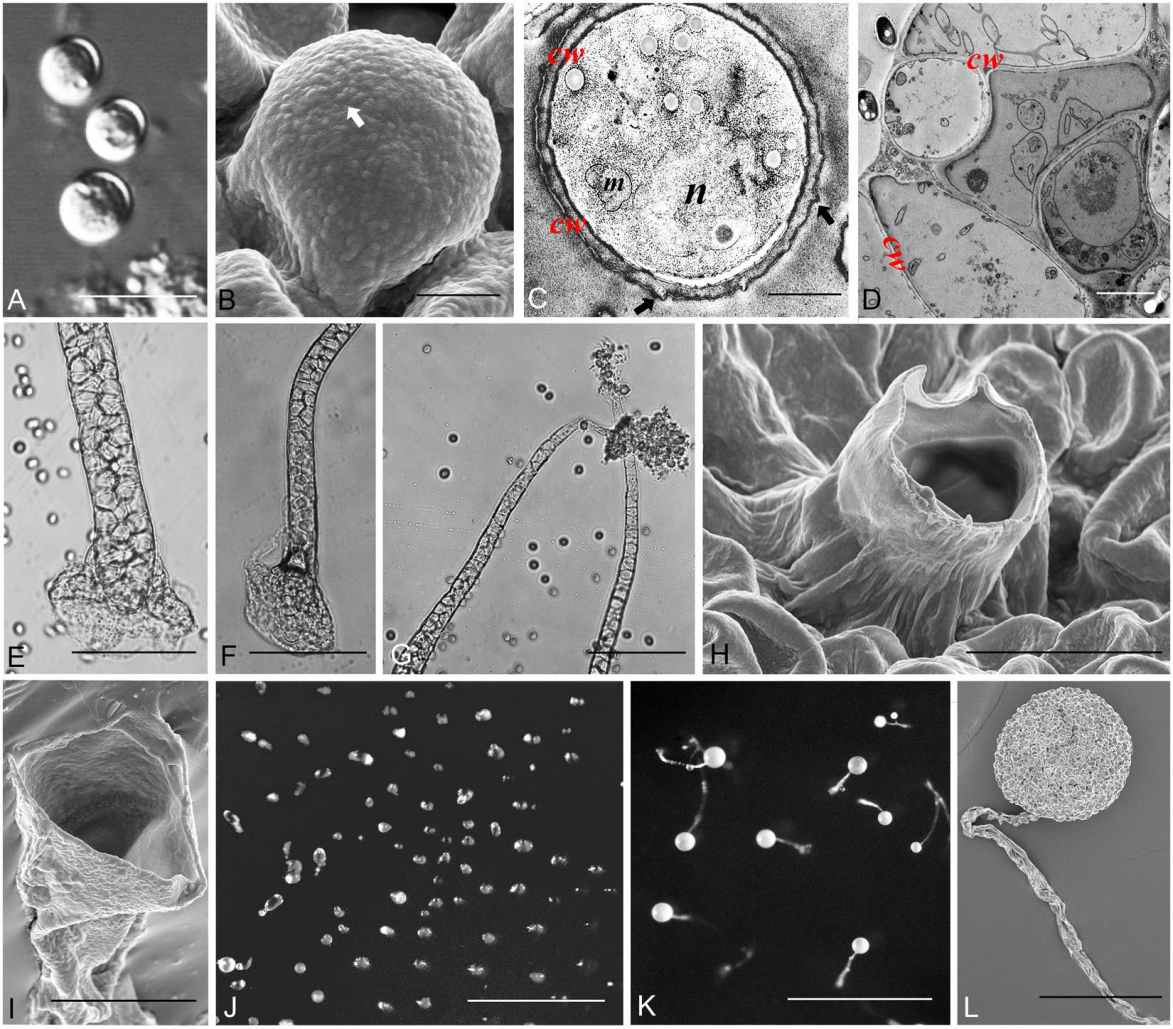
Morphological features of *Dictyostelium minimum*. (**A**) Spores; (**B**) Spores (SEM), arrow refers to globose bulges; (**C**) Spore (TEM), arrows refer to the uneven cell wall; (**D**) Cells of sorophore (TEM); (**E,F**) Base of sorophore; (**G**) Tips of sorophores; (**H,I**) Sori and tip of sorophore (SEM); (**J**) Aggregations; (**K**) Sorocarps; (**L**) Sorocarp (SEM). Abbreviations: n, nucleus; m, mitochondria; cw, cell wall. Bars: A = 10 µm; B,D = 2 µm; C = 0.5 µm; E,F,G = 50 µm; H = 5 µm; I = 4 µm; J = 500 µm; K = 1 mm; L = 50 µm.

**Figure 3:**
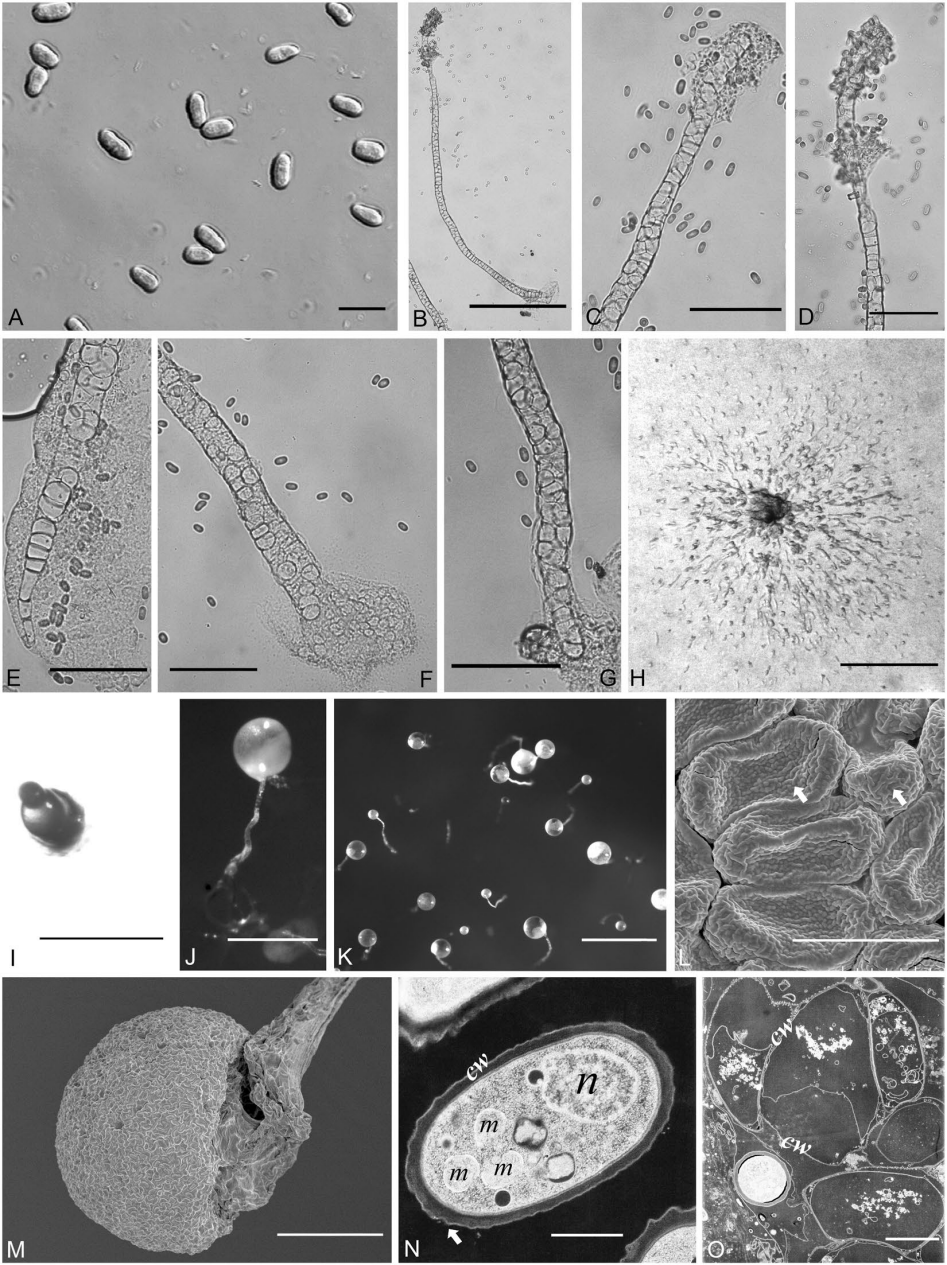
Morphological features of *Dictyostelium multiforme*. (**A**) Spores; (**B**) Sorophore; (**C,D**) Tip of sorophores; (**E,F,G**) Base of sorophore; (**H**) Aggregations; (**I**) Sorogen (**J,K**) Sorocarps; (**L**) Spores (SEM), arrows refer to ridges of spore surface; (**M**) Sorus (SEM); (**N**) Spore (TEM), arrow refers to the uneven cell wall; (**O**) Cells of sorophore (TEM). Abbreviations: n, nucleus; m, mitochondria; cw, cell wall. Bars: A = 10 µm; B = 200 µm; C,D,E,F,G = 50 µm; H,I = 200 µm; J = 500 µm; K = 1 mm; L = 4 µm; M = 50 µm; N = 833 nm; O = 2.5 µm.

**Figure 4:**
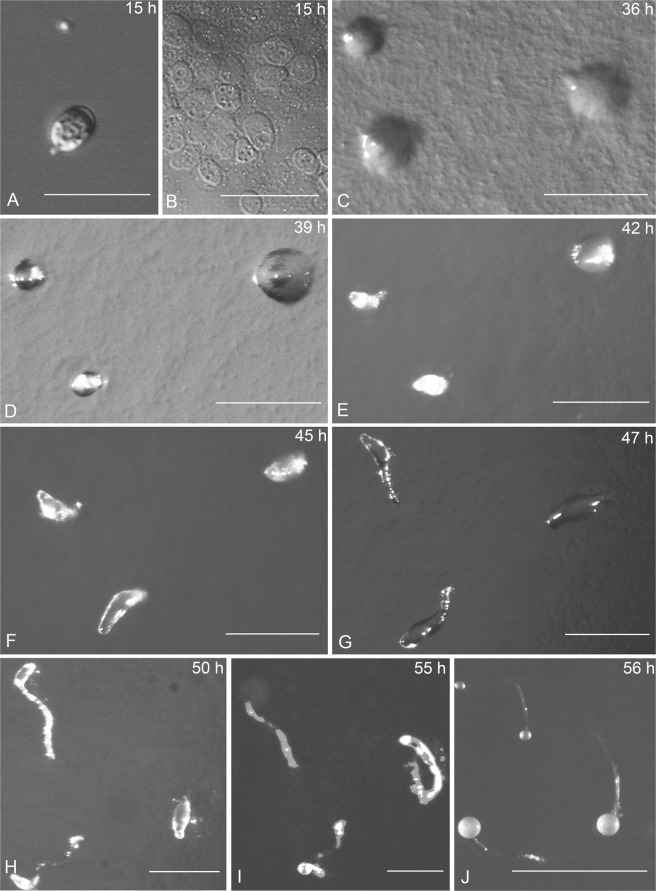
Life cycle of *Dictyostelium minimum*. The time of each stage showed on the top right corner. (**A**) Germinating spore; (**B**) Myxamoebae. (**C,D**) Aggregations; (**E–G**) Pseudoplasmodia; (**H,I**) Sorogen ascent; (**J**) Sorocarps. Bars: A = 10 µm; B = 50 µm; C,D,E,F,G,H,I = 200 µm; J = 1 mm.

**Figure 5:**
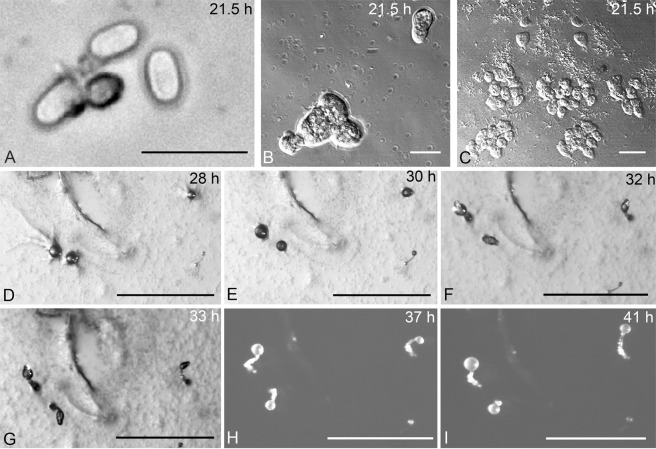
Life cycle of *Dictyostelium multiforme*. The time of each stage showed on the top right corner. (**A**) Germinating spore; (**B,C**) Myxamoebae; (**D,E**) Aggregations; (**F,G**) Pseudoplasmodia; (**H,I**) Sorocarps. Bars: A = 50 µm; B = 10 µm; C = 20 µm; D,E,F,G,H,I = 500 µm.

**Figure 6:**
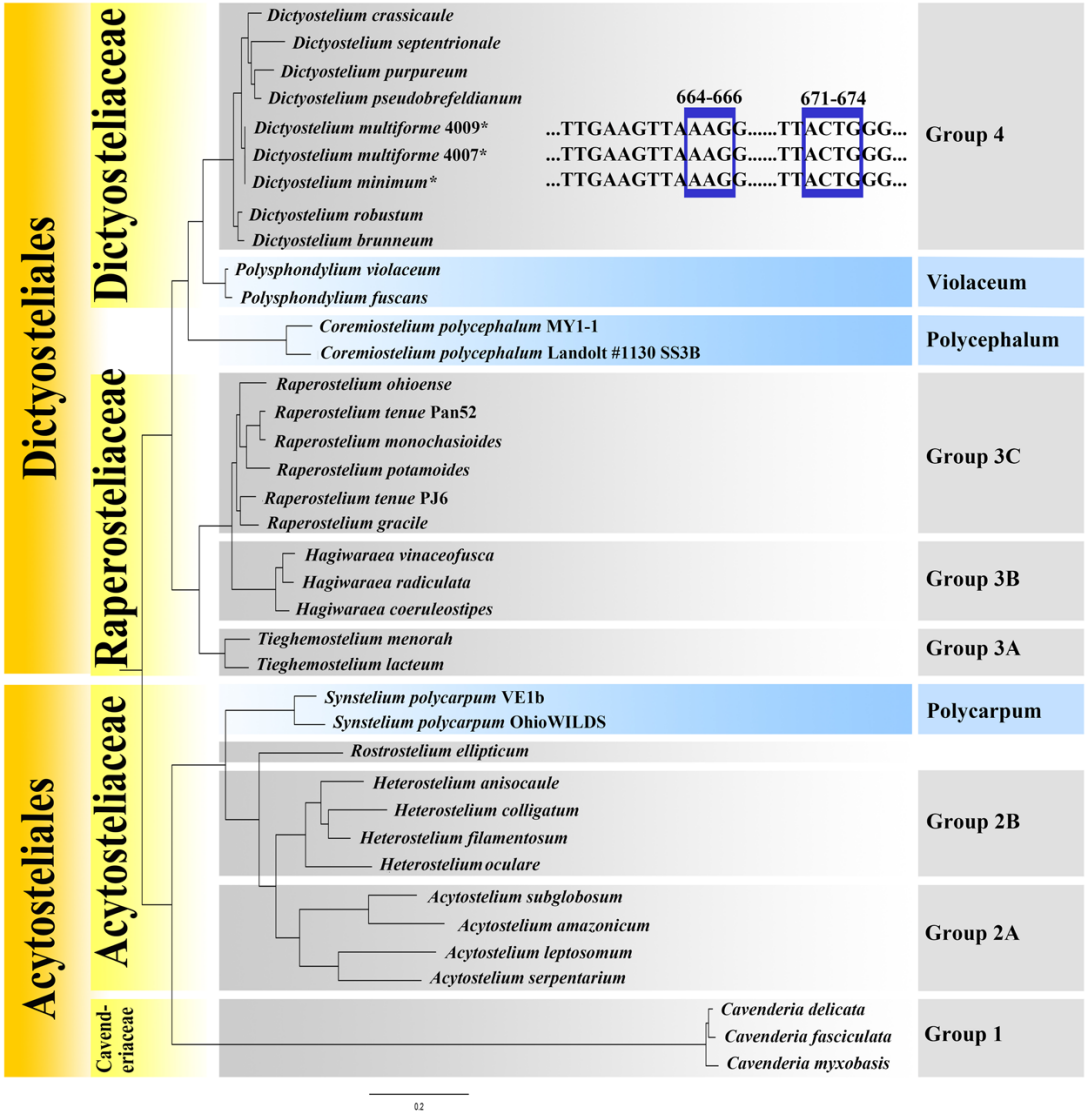
Phylogenetic tree of dictyostelids based on SSU rRNA and portions of the SSU rRNA gene alignment, showing molecular signatures of *Dictyostelium minimum* and *D. multiforme*. Newly generated sequences are indicated with asterisks.

After being processed, the samples collected from the Qinghai-Tibet Plateau yielded 16 isolates representing 12 species of dictyostelids (Fig. 1, Table S1). Three of these (*Dictyostelium brevicaule* Vadell & Cavender, *D. vermiforme* Vadell & Cavender, *Cavenderia fasciculata* [F. Traub er al.] S.Sheikh, Thulin & S.Baldauf) were reported for the first time from China. Six of these (*D*. *brefeldianum* H.Hagiw., *D. crassicaule* H.Hagiw., *C. antarctica* [Cavender *et al*.] S.Sheikh, Thulin & S.Baldauf, *C. aureostipes* [Cavender] S.Sheikh, Thulin & S.Baldauf, *C. exigua* (H.Hagiw.) S.Sheikh, Thulin & S.Baldauf, and *Heterostelium tikalense* [Vadell & Cavender] S.Sheikh, Thulin & S.Baldauf) are new records for the Qinghai-Tibet Plateau. Five of the six species (*D. crassicaule*, C*. antarctica*, *C. aureostipes*, *C. exigua*, and *H. tikalense*) had been recorded from China from only one previous record in Guizhou Province, Hubei Province, Liaoning Province, Taiwan Province and Jilin Province, respectively. The remaining two isolates are species new to science, and these have been given the names *D. minimum* and *D. multiforme* (Figs 2–5). Phylogenetic studies of the nuclear ribosomal small subunit  (SSU) rDNA showed the two new species *D. minimum* and *D. multiforme* are members of Group 4 (Fig. 6, Table S2), based on the concepts of Schaap *et al*.^19^, Romeralo *et al*.^20^, and Sheikh *et al*.^21^.

### Taxonomy and molecular phylogeny

*Dictyostelium minimum* Li Y., P. Liu et Y. Zou, sp. nov. (Fig. 2)

### MycoBank: MB823443

When cultured at 17 C on non-nutrient agar with *Escherichia coli*, sorocarps white, erect, stout, very short, only 95–720 μm high (average 324 μm), solitary, non-phototropic. Sorophore stout, tapering from base to tip, usually consisting single tier of cells from the middle portion to the tip, consisting of two or several tiers of cells from the middle portion to the base, base clavate or acuminate with accessory structures and two or several tiers of cells. The interior of the sorophore is hollow according to the SEM observations of the sorophore. Sori white, globose or citriform, 33–148 μm (average 72 μm) diam. Spores globose, without polar granules, 3.7–5.4 μm diam. Cell aggregations mound-like, without radiate streams, usually 39–73 × 29–57 μm (average 52 × 42 μm). Pseudoplasmodia not migrating without sorophore formation.

#### Etymology

This name refers to the small size of the sorocarps.

#### Holotype

HMJAU MR244. Isolated in 2012 (Strain 2794) from a soil sample collected from a mixed forest of *Picea asperata*, *Pinus densata*, *Quercus semecarpifolia*, *Pterocarya stenoptera*, and *Betula delavayi*, located at an elevation of approximately 3100 m in Lulang, Tibet.

#### Known distribution

Currently known only from China.

#### Commentary

There are primarily five species of dictyostelids that have globose spores^42^. These are *Dictyostelium rosarium* Raper & Cavender, *D. globisporum* Yu Li & P.Liu, *Raperostelium ibericum* (Romeralo *et al*.) S.Sheikh, *Tieghemostelium lacteum* (Tiegh.) S.Sheikh, Thulin & S.Baldauf var. *papilloideum* (Cavender) S.Sheikh, Thulin & S.Baldauf, and *T. lacteum* (Tiegh.) S.Sheikh, Thulin & S.Baldauf var. *papilloideum* (Cavender) S.Baldauf, S.Sheikh & Thulin. The spores of *D. globisporum*, *R. ibericum*, and *T. lacteum* all have polar granules, which are lacking in *D. minimum*. Although *D. rosarium* has inconspicuous polar granules, the aggregations in this species have radiate streams and form clustered sorocarps. The sorocarps of *T. lacteum* var. *papilloideum* (0.6–1.4 mm) are larger than those of *D. minimum*, the sorophores are thinner than those of *D. minimum*, and the pseudoplasmodia of *T. lacteum* var. *papilloideum* migrate without sorophore formation.

**Figure 7:**
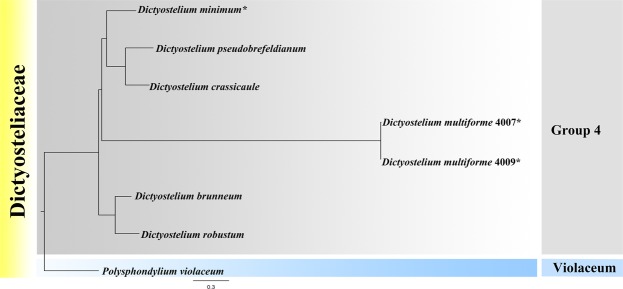
Phylogenetic tree of dictyostelid Group 4 species based on ITS rDNA. Newly generated sequences are indicated with asterisks.

This species belongs to Dictyostelid Group 4^19–21^ in SSU rDNA phylogeny (Fig. 6). It forms a clade together with *D. multiforme*. In the ITS phylogenetic tree (Fig. 7), *D*. *minimum* forms a clade with *D. crassicaule* and *D. pseudobrefeldianum* H.Hagiw. However, the most noteworthy difference between them is the presence of globose spores in *D. minimum*. The SSU and ITS rDNA sequences for *D. minimum* are available at GenBank, with the accession numbers MG490369 and MG490372, respectively.

*Dictyostelium multiforme* Li Y., P. Liu et Y. Zou, sp. nov. (Fig. 3)

### MycoBank: MB823442

When cultured at 23 C on non-nutrient agar with *E. coli*, sorocarps white, solitary or gregarious, erect or semi-erect, without branches or rarely with one branch. Semi-erect sorocarps mostly 0.68–1.80 mm long, sometimes with sinuous sorophores which have an “S” shape in the central portion, other semi-erect sorophores straight, consisting of several tiers of cells. Erect sorocarps mostly 0.21–1.13 mm long, sorophores straight, consisting one or two tiers of cells. Sorophore tips clavate, mostly 12.0–26.9 µm, with two or several tiers of cells, semi-erect, the tips with dense refractile matrices of slime present. Sorophore base clavate, acuminate, or conical, mostly 9.4–20.1 µm, with one or two tiers of cells, with basal support disk. The interior of the sorophore is hollow according to the SEM observations of the sorophore. Sori white, globose, mostly 90–250 µm diam. Spores oblong or elliptical, sometimes reniform, without polar granules, mostly 5.8–12.6 × 3.6–5.0 µm. Cell aggregations mound-like, minutum-type, without radiate streams. Pseudoplasmodia not migrating without sorophore formation.

#### Etymology

This name of the species refers to the presence of several types of sorocarps.

#### Holotype

HMJAU MR245. Isolated in 2013 (Strain 4007, 4009) from a sample of animal dung, the sample collected at an elevation of 4275 m in Yushu, Qinghai Province.

#### Known distribution

Currently known only from China.

#### Commentary

This species is similar to *Dictyostelium barbibulus* Perrigo & Romeralo*, D. microsorocarpum* Yu Li & Xiao L.He, *D. mucoroides* Bref. var. *stoloniferum* Cavender & Raper, and *D. crassicaule*, with the significant differences among them discussed below. However, the sorophore base of *D. barbibulus* is 50–80 µm, which is wider than that of *D. multiforme*. Moreover, the sorocarp of *D. barbibulus* is erect. The aggregation of *D. microsorocarpum* is radiate, whereas the aggregation of the new species is mound-like, and the sori of *D. microsorocarpum* are smaller than those of the new species. The sorocarps of *D. mucoroides* var. *stoloniferum* are phototropic, while the new species does not have this feature. The aggregations of *D. crassicaule* are radiate and the sorophore tips are capitate, whereas *D. multiforme* has mound-like aggregations, clavate sorophore tips and more narrow sorophore bases than is the case for *D. crassicaule*.

This species belongs to Dictyostelid Group 4^19–21^ in the SSU rDNA phylogeny (Fig. 6). It forms a clade along with *D. minimum*. Morphologically, the spore shape and size, sorophore cell tiers and sorocarp appearance are different between *D. multiforme* and *D. minimum*. In the ITS phylogenetic tree (Fig. 7), *D. multiforme* is in a different clade with *D. minimum* which form a single clade in Group 4. The SSU and ITS rDNA sequences for *D. multiforme* are available at GenBank, the accession numbers are MG490370 (Strain 4007), MG490371 (Strain 4009), MG490373 (Strain 4007,) and MG490374 (Strain 4009), respectively.

### Ontogeny

#### Life cycle of *Dictyostelium minimum*

Spore germination (Fig. 4A) begins with the appearance of a minute pore dissolved in the spore wall after 15 h on agar. At the same time, several myxamoebae (Fig. 4B) are released from the spores. Myxamoebae are colorless, transparent, and irregular. When a “critical mass” of myxamoebae occurs, myxamoebae aggregate to one center to form a single aggregation (Fig. 4C), with no cell streams 36 h after the spores have been inoculated on agar. The aggregations (Fig. 4D) grow larger and rise up in the center after 39 h. The aggregations culminate (Fig. 4E) and began to form pseudoplasmodia with indistinct sorophores 42 h after inoculation. The sorophore (Fig. 4F) begins to form and grow longer at about 45 h. Two hours later, the slug (Fig. 4G) begins to form and with no movement in this process. Three hours later, the sorogen is produced and is prostrate on the surface of the agar, and the young sorocarp begins to fruit (Fig. 4H). The top of the sorogen became tapered, then changes to globose, and the sorophore becames longer and curved on the agar (Fig. 3I). Fifty-six h after inoculation, the sorocarps (Fig. 4J) fruit; the sorocarps are very small, solitary, and non-phototropic.

The entire life cycle of *D. minimum* extends over 3 d. The spores germinated and released myxamoebae 15 h after inoculation. The aggregations formed 36 h after inoculation. Pseudoplasmodia formation required 42 h. Then, eight hours later, the sorogen formed. After having been inoculated 56 h previously, the sorocarps fruited.

#### Life cycle of *Dictyostelium multiforme*

Elliptical spore germination (Fig. 5A) begins with the dissolution of the spore wall to release myxamoebae (Fig. 5B,C) after 21.5 h on agar. Myxamoebae are colorless, transparent, and irregular. Their shape changed as they moved about, feeding upon bacteria, growing larger and aggregating as a result of being attracted by cAMP to form a single mould-like aggregation (Fig. 5D) without radiated cell streams 28 h after the spores had been inoculated on agar. After the aggregating of more and more myxamoebae, the cell aggregations (Fig. 5E) rise up in the center begin to form pseudoplasmodia with indistinct sorophores 30 h after inoculation. Two hours later, the sorophores begin to form (Fig. 5F). Sorophores grow longer, which gives them the appearance of a fruiting sorophore (Fig. 5G) at 33 h, pseudoplasmodia form and undergo a very short period of movement. The sorophore is slightly curved, which gives it an “S” shape in the central portion. After the formation of the sorophore, the sori begin to grow gradually to form globose sori at 37 h (Fig. 5H). Forty-one h after inoculating spores on the agar, the sorocarps (Fig. 5I) finally fruit. Sorocarps are solitary, erect or semi-erect and lack branches.

The whole life cycle of *D. multiforme* extends of a period of less than 2 d (41 h). The spores germinated and released myxomoebae 21.5 h after inoculation. The aggregations formed 28 h after the inoculation, whereas the pseudoplasmodia formation needed 33 h. After that, the sorophores and sori begin to grow orderly and finally form fruiting sorocarps after 41 h. The formation of myxomoebae of this species is later than that of *D. minimum*; however, the formation of sorocarps from pseudoplasmodia in this species is shorter than that of *D. minimum*.

### Ecology

#### Dictyostelids-elevation relationships

**Figure 8:**
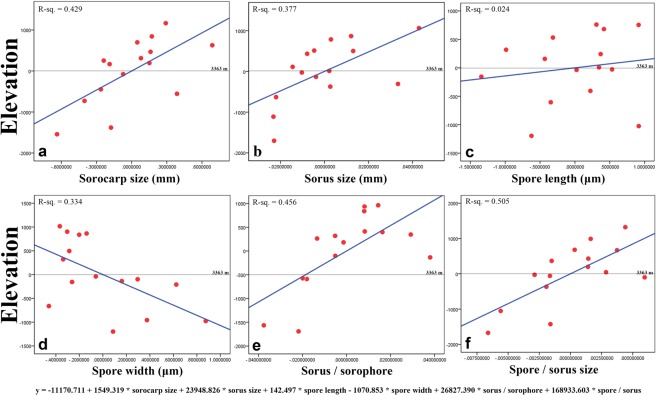
Correlation of morphological features of 11 species of *Dictyostelium* and *Cavenderia* (15 isolates in total in this paper) with elevation. R sequared values for the linear regression are given in each panel. (**A**) Sorocarp size; (**B**) Sorus size; (**C**) Spore length; (**D**) Spore width; (**E**) Ratio of sorus and sorophore; (**F**) Ratio of sorus and spore size. y = −11170.711 + 1549.319 * sorocarp size + 23948.826 * sorus size + 142.497 * spore length − 1070.853 * spore width + 26827.390 * sorus / sorophore + 168933.603 * spore / sorus. “y = 0” represents the average elevation of those isolates, 3363 m (grey lines). “x = 0” reprensents the average of each predictors. The blue lines represent the trend of each predictors with elevations. Increases in elevation led to increasing sorocarp size, sorus size, spore length, ratio of sorus and sorophore, and ratio of sorus and spore size. In contrast, increasing elevation was correlated with decreasing spore width.

Environmental factors such as elevation clearly have an effect on the biodiversity and morphological features of species of dictyostelids. Considering the 11 species (15 isolates) of dictyostelids isolated in the present study, except *Heterostelium tikalense*, all have traditional simple sorophore, so the relationships between morphological characteristics of those 15 isolates and their elevations were analyzed. From the results of linear correlation analysis of elevation with the six predictors considered in the present study, increases in elevation led to increasing sorocarp size, sorus size, spore length, ratio of sorus and sorophore, and ratio of sorus and spore size (Fig. 8A–C,E,F). In contrast, increasing elevation was correlated with decreasing spore width (Fig. 8D).

#### Dictyostelids-forest type relationships

In the present study, 13 isolates of dictyostelids were obtained from different forest types including mixed forest, broadleaf forest, coniferous forest, grassland, and alpine grassland. Another three isolates of dictyostelids were isolated from animal dung. After make an analysis of the morphological charactertistics of those dictyostelids with those forest types, we found there was no linear correlation of forest type with sorocarp size, sorus size, spore length, spore width, ratio of sorus and sorophore, and ratio of sorus and spore size.

## Discussion

### Effects of elevation on the biodiversity of dictyostelids

Ecological characteristics such as elevation clearly have an effect upon the biodiversity of dictyostelids. However, studies of the distribution and abundance of dictyostelids associated with similar habitats at high elevations (>2000 m) are exceedingly limited. Although Cavender *et al*.^12^ and Landolt *et al*.^10^ indicated that differences in elevation (<2000 m) and forest types affected the distribution patterns of dictyostelids in the Great Smoky Mountains National Park, a number of new species were recovered at high elevations in the park. These authors also commented on the apparent negative effect of elevation on dictyostelid abundance and a possible positive effect on species richness^43^. On the other hand, Paillet and Satre^7^ reported higher biodiversity of dictyostelids at higher elevations (<2000 m) in French Alps. The present study firstly investigated dictyostelid biodiversity in mountains at elevations >2000 m. Five species was recorded from samples collected at elevations of 2000–3000 m, four species at elevations of 3000–4000 m, and four species from samples collected at elevations of 4000–5000 m. This suggests that dictyostelids are probably not uncommon at higher elevations (>2000 m), although studies carried out at such elevations are exceedingly limited.

### Effects of elevation on the morphology of dictyostelids

Elevation also affected the morphological features of the species of dictyostelids being considered. There was a positive correlation of increasing of elevation with sorocarp size, sorus size, spore length, ratio of sorus and sorophore, and ratio of sorus and spore size, although the *R*^2^ values for linear correlations of spore length were weak (Fig. 8). Dictyostelids carry bacteria during spore dispersal and thus can “seed” a new food crop, which is a major advantage if edible bacteria are lacking at the new site^44^. Presumably, the larger the size of the sorocarp and sori would allow them carry more bacteria if edible bacteria are not abundant enough in habitats such as those found at higher elevations on the plateau.

### Substrate types

Dictyostelids have been recovered from animal dung^45^, forest soil^17^, grassland soil^46^, canopy soil^47,48^, and soil in an agricultural field^49^. In the present study, dictyostelids were recovered from both animal dung and forest soil. From these samples, four species (*Dictyostelium brevicaule*, *D. minimum*, *D. sphaerocephalum*, *Cavenderia aureostipes*) were recorded from mixed forest soil, three species (*D. vermiforme*, *D. sphaerocephalum*, C*. fasciculata*) from coniferous forest soil, three species (*D. sphaerocephalum*, *C. exigua*, *Heterostelium tikalense*) from broadleaf forest soil, two species (*D. brefeldianum*, *D. sphaerocephalum*) from alpine grassland soil, one species (*C. antarctica*) from grassland soil, and two species (*D. crassicaule*, *D. multiforme*) from animal dung. However, there were no positive linear correlations of forest type with sorocarp size, sorus size, spore size, ratio of sorus and sorophore, and ratio of sorus and spore size. There are the highest genus diversity and species diversity of dictyostelids in broadleaf forest and mixed forest seperately in this study.

### Environmental conditions affect the distribution of dictyostelids

*Dictyostelium minimum* has very small sorocarps, and the sorophore is stout and thick, which is a character also found in species which occur in habitats characterized by cool temperatures (20 C or lower) in order to keep the sorocarp erect during slow development^17^. Presumably, the small size of the sorocarps would allow them to compete more successfully under conditions that are marginal for larger species, such as those microhabitats with a limited bacterial food supply^10^. In the present study, we used both 17 C and 23 C to culture the new species *D. minimum*; however, it grew well at 17 C and but was unstable and sometimes even unsuccessful at 23 C.

*Dictyostelium sphaerocephalum* tends to be more abundant in habitats where conditions are less favorable^50–52^. Examples include extremely cool, dry, or disturbed habitats^15^ such as in the tundra^50,53^. In our study, *D. sphaerocephalum* was also recovered at three collecting sites located at higher elevations (3000–4600 m). The occurrence of this species in alpine grassland soil, mixed forest soil and coniferous forest soil provide additional data to support the concept that *D. sphaerocephalum* is a truly widespread species^7^.

In contrast, *D. crassicaule*, *C. exigua*, and *C. fasciculata* were reported as restricted species in soils of the world’s forests^43^, herein they were all found again from forest soil and animal dung, which supports the point of view^43^ that animal vectors and plant associations have a major role in determining the distribution of dictyostelids.

Futhermore, the highest relative abundance of dictyostelids in this study are Localities (ii) Medog and (v) Lulang (Fig. 1). We found Medog has the highest annual temperature, and Lulang has the highest annual precipitation within localities of this study. Presumably, the species abundance of dictyostelids correlated with the environmental conditions such as temperature and precipitation a lot.

### Ultrastructure of the two new species

From SEM and TEM images obtained for *D. minimum* and *D. multiforme*, we found that the spore wall was characterized by the presence of small bulges. The bulges of *D. minimum* are nearly globose and short. The ridged bulges of *D. multiforme* are deeper and longer than those of *D. minimum*. These data suggest that the surface of dictyostelid spore may differ with respect to ornamentation.

### Molecular phylogeny

Detailed analysis involving the alignment of SSU of these two new species *D. minimum* and *D. multiforme* indicated that AAG and ACTG occur in the nucleotide positions 664–666 and 671–674, respectively (Fig. 6); this indicates that they clearly belong to genus *Dictyostelium*^21^. However, the globose spores of *D. minimum* add a new spore feature to be considered in the new classification of *Dictyostelium*. In the SSU rDNA phylogenetic tree (Fig. 6), *D. minimum* and *D. multiforme* were in the same clade. However, when the phylogenetic relationships of Group 4 species were analyzed with the ITS rDNA (Fig. 7), *D. minimum* and *D. multiforme* occurred in different clades.

In the SSU phylogenetic tree published by Sheikh *et al*.^21^, *Synstelium* was not assigned to any family or order; herein we found it was most closely related to clade Acytosteliaceae in the Acytosteliales. Consequently, *Synstelium* was recognized in the Acytosteliaceae and Acytosetliales in the present study. All other taxonomic levels (genus, family and order) are consistent with those given by Sheikh *et al*.^21^.

Three species (*D. rosarium*, *Tieghemostelium lacteum*, and *Raperostelium ibericum*) with globose spores are members of Group 4, Group 3 A, and Group 3 C, respectively^21^. In the present study, another species (*D. minimum*) with globose spores was obtained which belongs to Group 4 according to the new classification of Sheikh *et al*.^21^. These four globose spore speices all belongs to Dictyostliales of two families (Group 3 and Group 4). As such, it is not possible to use only a single morphology-based taxonomic feature such as spore shape to differentiate species in the dictyostelids.

## Materials and Methods

### Sampling

Samples used for isolation of dictyostelids were collected from nine sites on the Qinghai-Tibet Plateau in China during the period of 2012, 2013 and 2016. A total of 114 samples, including soil and animal dung, each approximately 30–50 g, were collected and placed in sterile whirl-pack plastic bags. In most instances, at least five samples were collected from each vegetation type at each locality. Afterwards, these samples were returned to the laboratory as soon as possible, following the recommendations of Cavender and Raper^5^. Each sample bag was numbered and the sample itself preserved at 4 C in the herbarium of the Mycological Institute of Jilin Agricultural University (HMJAU), Changchun, China.

Localities (Fig. 1, Figure S1) where samples were collected on the Qinghai-Tibet Plateau were (i) the Sejila Mountain National Forest Park, Nyingchi, Tibet, average elevation 3100 m, annual precipitation 650 mm, annual temperature 8.7 C; (ii) Medog, Tibet, a moist region, average elevation 1200 m, annual precipitation 900 mm, annual temperature 16 C; (iii) Bome, Tibet, elevation 2003–6648 m, annual precipitation 977 mm, annual temperature 8.5 C; (iv) Zayu, Tibet, elevation 1400–6740 m, annual precipitation 801 mm, annual temperature 12 C; (v) Lulang, Tibet, average elevation 3700 m, annual precipitation 1849 mm, annual temperature 12 C; (vi) Lhasa, Tibet, average elevation 3650 m, annual precipitation 200–510 mm, annual temperature 7.4 C; (vii) Yushu, Qinghai Province, average elevation 4200 m, annual precipitation 487 mm, annual temperature 2.9 C; (viii) Huzhu Beishan National Forest Park, Qinghai Province, average elevation 2100–4308 m, annual precipitation 477 mm, annual temperature 5.8 C; and (ix) the Jiuzhaigou National Forest Park, Aba, Sichuan Province, average elevation 2050–4964 m, annual precipitation 836 mm, annual temperature 6.1 C.

### Isolation and cultivation

The isolation methods used in the present study followed those described by Cavender and Raper^5^. Each sample was weighed and diluted for an initial dilution of 1:10 by adding ddH_2_O. This dilution was shaken to disperse the material and to suspend the amoebae and spores of dictyostelids. Afterwards, a 0.5 mL aliquot of this dilution was added to each of five duplicate culture plates prepared with hay infusion agar^2^. Approximately 0.4 mL of a heavy suspension of the bacterium *E. coli* was added to each culture plate as a food source. The plates were incubated at temperatures of 17 and 23 C with a 12 h light and dark cycle. Each plate was examined at least once a day for two weeks after the appearance of initial aggregations. Each isolate was purified and cultivated for taxonomic studies and preservation on non-nutrient water agar plates with *E. coli* pregrown for 12–24 h. Spores from these plates were frozen in HL 5 media^54^ and stored at −80 C in HMJAU, Changchun, China.

### Morphological features and life cycle observations

Dictyostelid isolates were identified with the use of the descriptions provided by Raper^2^, whose nomenclature also was followed except for those species recently assigned to new genera in the system of classification proposed by Sheikh *et al*.^21^. In the primary isolation plates, the locations of each early aggregating clone and sorocarp that developed were marked. The characteristic stages in the life cycle, including cell aggregation and the formation of pseudoplasmodia, and sorocarps were observed under a Zeiss dissecting microscope (Axio Zoom V16) with a 1.5× objective and 10× ocular. Slides with sorocarps were prepared with water as the mounting medium. Features of spores, sorophores, and sorocarps were observed and measured on the slides by using a Zeiss light microscope (Axio Imager A2), with 10× ocular and 10, 40, and 100× (oil) objectives. Photographs were taken with Zeiss Axiocam 506 color microscope camera.

#### Observation of spore germination

Hanging drop cultures as described by Keller and Schoknecht^55^ were prepared for the observation of spore germination. Spores obtained from a sorus were mixed with a droplet of sterile water on the undersurface of a 22-mm square cover glass. The cover glass was then inverted over a depression slide. Vaseline was used to ring the edges of the cover glass. Spores were freely suspended in the water droplet. Features of the myxamoebae were observed and photographed by a Zeiss laser confocal microscope (LSM 710).

#### Scanning electron microscopy

Spores of new species *Dictyostelium minimum* and *D. multiforme* were prepared for scanning electron microscopy according to the method of Boyde and Wood^56^. The collected spores were washed, fixed, dehydrated, dried, and prepared for study, then observed by with Hitachi scanning electron microscope (SU 8010).

#### Transmission electron microscopy

The sorocarps of new species *D. minimum* and *D. multiforme* were prepared for transmission electron microscopy according to standard techniques^57^. The sorocarps were collected with a prefixation in 4% glutaraldehyde for more than 4 h at 4 C, followed by postfixation in 2% osmium tetroxide for 2 h at 4 C, with both fixatives buffered in 0.05 N Na-cacodylate buffer at pH 7.4. Afterwards, the samples were dehydrated in water, ethanol and acetone, embedded in SPI-PON 812 for 12 h at 35 C, for 12 h at 45 C, and for 24 h at 60 C. After these processes had been completed, the sections to be observed were cut on a Leica EM UC7, stained in uranyl acetate and lead citrate, observed and photographed by a Hitachi transmission electron microscope (H-7650).

### DNA isolation, PCR amplification and sequencing

After amoebae had cleared *E. coli* on the water agar media, the spores of dictyostelid isolates to be studied were collected with a sterile tip, then those spores were mixed with the lysed buffer of the NuClean Plant Genomic DNA Extraction Kit from CW Biotech (Beijing, China) and the following steps were carried out according to the instructions provided along with this kit. The genomic DNA solution was used directly for the PCR amplification. The SSU and ITS rDNA markers were amplified using the primers 18SF–A and 18SR–B, D542F and D1340R, and ITS1 and ITS4 (Table S3). PCR products were sent to Sangon Biotech Co., Ltd. (Shanghai) for sequencing.

### Phylogenetic analysis

The newly-generated sequences were checked and then submitted to GenBank. Sequences for all closely related species were downloaded from GenBank (Table S2) for phylogenetic analysis. The ITS and SSU sequences were aligned and compared separately using the program Muscle v.3.6^58,59^, then manually adjusted in MEGA 7.0^60^. Maximum likelihood (ML) analyses were performed using RAxML v7^61^. In the ML analyses, the best-fit substitution models were estimated using GTR submission model and a gamma correction for rate variation among sites (GTRGAMMA), using the CIPRES server. The statistical support of clades was assessed with 1000 rapid-bootstrap (BS) replications.

### Ecological statistics analysis

Sorocarp size, sorus size, spore length, spore width, ratio of sorus and sorophore, and ratio of sorus and spore size data for the 11 species of *Dictyostelium* and *Cavenderia* being considered (15 isolates in total) in this study were inputted to the IBM SPSS 19.0 version software as predictors for linear regression analysis of those six predictors (sorocarp size, sorus size, spore length, spore width, ratio of sorus and sorophore, and ratio of sorus and spore size) with the two dependent variables of elevation and forest type, with each of the latter considered separately.

### Nomenclature

According to the International Code of Nomenclature for algae, fungi, and plants, the electronic version of this article in Portable Document Format (PDF) will represent a published work. In addition, new names contained in this study have been submitted to MycoBank and will each be allocated a unique MycoBank number which will be accessible through MycoBank, Index Fungorum, GBIF and other international biodiversity initiatives where they will be made available to the Global Names Index.

## Supplementary information

Supplementary Information
