# Supplementary material for: Two new species of dictyostelid cellular slime molds in high-elevation habitats on the Qinghai-Tibet Plateau, China
Source: Sci Rep. 2019 Jan 9;9:5. doi: 10.1038/s41598-018-37896-7 (PMC6327048; doi:10.1038/s41598-018-37896-7)
Supplement: Supplementary file 1 — Supplementary Information [file 41598_2018_37896_MOESM1_ESM.doc]

**Two new species of dictyostelid cellular slime molds in high-elevation habitats on the Qinghai-Tibet Plateau, China**

**Pu Liu^1^, Yue Zou^1^, Shu Li^1^, Steven L. Stephenson^2^, Qi Wang^1^*, Yu Li^1^***

^1^ Engineering Research Center of Chinese Ministry of Education for Edible and Medicinal Fungi, Jilin Agricultural University, Changchun, 130118, P. R. China,

^2^ Department of Biological Sciences, University of Arkansas, Fayetteville, AR 72701, USA

*** Qi Wang and Yu Li** are the corresponding authors for the manuscript (qwang2003@hotmail.com, [yuli966@126.com](mailto:yuli966@126.com,)).

Pu Liu is only for all the correspondence for all stages (puliu1982@yahoo.com, phone: 86-13500812634, fax: 86-43184510966).

References:

1. Medlin L, Elwood HJ, Stickel S, Sogin M. The characterization of enzymatically amplified eukaryotic 16S-like rRNA-coding regions. Gene **71 (2)**, 491-499 (1988).
2. Schaap, P. *et al*. Molecular phylogeny and evolution of morphology in the social amoebas. *Science* **314**, 661*–*663 (2006).
3. White T.J., T. Bruns, S. Lee, et al. Amplification and direct sequencing of fungal ribosomal RNA genes for phylogenetics. In: Innis MA, Gelfand DH, Sninsky JJ et al // PCR Protocols: A Guide to Methods and Applications. California, San Diego, 315–322 (1990).
4. Romeralo, M., Landolt, J. C., Cavender, J. C., Laursen, G. A. & Baldauf, S. L. Two new species of dictyostelid cellular slime molds from Alaska. *Mycologia* **102**, 588*–*595 (2010).

**Supplementary Table S1** List of species isolated from samples collected on the Qinghai-Tibet Plateau in the present study.

| Species | Strain No. | Locality | Coordinates | Elevation  (m) | Habitat (with the major plant taxa present given in parentheses) |
| --- | --- | --- | --- | --- | --- |
| *Cavenderia antarctica** | 3013 | Huzhu Beishan National Forest Park, Qinghai | 36.50.44.49N, 101.56.44.07E | 2300 | grassland soil |
| *C. aureostipes** | 2791 | Bome, Tibet | 29.51.27.22N, 95.46.1.76E | 2730 | mixed forest soil  (*Picea asperata*, *Pinus densata*, *Pinus armandii*, *Quercus semecarpifolia*, *Platycladus orientalis*, *Pterocarya stenoptera*, and *Betula delavayi*) |
| *C. exigua** | 4029 | Zayu, Tibet | 28.35.11.83N, 95.42.58.15E | 1000 | broadleaf forest soil  (*Pterocarya stenoptera*, *Betula delavayi*, and *Platycladus orientalis*) |
| *C. fasciculata*** | 5239 | Jiuzhaigou National Forest Park, Sichuan | 32.45.4.75N, 103.37.14.22E | 3330 | coniferous forest soil  (*Picea asperata* and *Abies fabri*) |
| *Dictyostelium brefeldianum** | 4033 | Zayu, Tibet | 29.33.20.96N, 96.26.59.32E | 4100 | alpine grassland soil  (*Polygonum viviparum*, *Rhododendron simsii*, *Artemisia* sp., *Carex* sp., *Oxytropis* sp., and *Rosa* sp.) |
| *D. brevicaule*** | 5255 | Jiuzhaigou National Forest Park, Sichuan | 33.04.8.77N, 103.51.27.30E | 2820 | mixed forest soil  (*Acer saccharum*, *Betulaceae delavayi*, *Abies fabri*, *Tsuga chinensis*, *Picea asperata*, and *Pinus tabuliformis*) |
| *D. crassicaule** | 4008 | Yushu, Qinghai | 38.24.36.50N, 97.18.14.72E | 4275 | animal dung (dog) |
| *D. minimum**** | 2794 | Lulang, Tibet | 29.41.46.47N, 94.43.43.16E | 3100 | mixed forest soil  (*Picea asperata*, *Pinus densata*, *Quercus semecarpifolia*, *Pterocarya stenoptera*, and *Betula delavayi*) |
| *D. multiforme**** | 4007 | Yushu, Qinghai | 38.24.36.50N, 97.18.14.72E | 4275 | animal dung (dog) |
|  | 4009 | Yushu, Qinghai | 38.24.36.50N, 97.18.14.72E | 4275 | animal dung (dog) |
| *D. sphaerocephalum* | 2795 | Bome, Tibet | 29.51.42.49N, 95.46.0.38E | 3000 | broadleaf forest soil (*Carya cathayensis*) |
|  | 4023 | Sejila Mountain National Forest Park, Nyingchi, Tibet | 29.36.40.32N, 94.39.6.21E | 4517 | alpine grassland soil  (*Rhododendron simsii*) |
|  | 4036 | Bome, Tibet | 29.45.19.35N, 95.56.25.12E | 3400 | mixed forest soil  (*Picea asperata*, *Pinus densata*, *Pinus armandii*, *Quercus semecarpifolia*, *Platycladus orientalis*, *Pterocarya stenoptera*, and *Betula delavayi*) |
|  | 4027 | Medog, Tibet | 29.49.43.81N, 95.42.58.15E | 3700 | coniferous forest soil  (*Picea asperata* and *Pinus densata*) |
| *D. vermiforme*** | 3005 | Huzhu Beishan National Forest Park, Qinghai | 36.48.57.37N, 102.31.26.09E | 2300 | coniferous forest soil  (*Platycladus orientalis*) |
| *Heterostelium tikalense** | 2793 | Lhasa, Tibet | 29.39.51.05N, 91.03.51.94E | 3645 | broadleaf forest soil  (*Pterocarya stenoptera*) |

* Refers to new records for the Qinghai-Tibet Plateau

** Refers to species new to China

*** Refers to species new to science

**Supplementary Table S2** NCBI GenBank accession information for sequences of all dictyostelids included in the phylogenetic analysis. Newly generated sequences are indicated with asterisks.

| Taxon | Isolate no. | Accession no. | Gene |
| --- | --- | --- | --- |
| *Acytostelium amazonicum* | HN1B1 | HQ141511.1 | SSU |
| *A. leptosomum* | FG12 | AM168111.1 | SSU |
| *A. serpentarium* | SAB3A | AM168113.1 | SSU |
| *A. subglobosum* | LB1 | AM168110.1 | SSU |
| *Cavenderia delicata* | TNS-C-226 | AM168093.1 | SSU |
| *C. fasciculata* | SH3 | AM168087.1 | SSU |
| *C. myxobasis* | NT2A | HQ141522.1 | SSU |
| *Coremiostelium polycephalum* | MY1-1 | AM168056.1 | SSU |
| *C. polycephalum* | [Landolt #1130 SS3B](https://www.ncbi.nlm.nih.gov/nuccore/HQ141488.1/) | HQ141488.1 | SSU |
| *Dictyostelium brunneum* | WS700 | AM168031.1 | SSU |
| *D. brunneum* | WS700 | HQ141451.1 | ITS |
| *D. crassicaule* | 93HO-33 | AM168037.1 | SSU |
| *D. crassicaule* | 93H033 | HQ141456.1 | ITS |
| *D. minimum** | 2794 | MG490369.1 | SSU |
| *D. minimum** | 2794 | MG490372.1 | ITS |
| *D. multiforme** | 4009 | MG490371.1 | SSU |
| *D. multiforme** | 4007 | MG490370.1 | SSU |
| *D. multiforme** | 4007 | MG490373.1 | ITS |
| *D. multiforme** | 4009 | MG490374.1 | ITS |
| *D. pseudobrefeldianum* | 91HO8 | AM168059.1 | SSU |
| *D. pseudobrefeldianum* | 91HO8 | HQ141470.1 | ITS |
| *D. purpureum* | C143 | AM168060.1 | SSU |
| *D. robustum* | TNS-C-219 | AM168064.1 | SSU |
| *D. robustum* | Ch53 | HQ141473.1 | ITS |
| *D. septentrionale* | IY49 | AM168066.1 | SSU |
| *Hagiwaraea coeruleostipes* | CRLC53B | AM168036.1 | SSU |
| *H. radiculata* | ML5A | HQ141494.1 | SSU |
| *H. vinaceofusca* | CC4 | AM168062.1 | SSU |
| *Heterostelium anisocaule* | NZ47B | AM168096.1 | SSU |
| *H. colligatum* | HN13C1 | HQ141505.1 | SSU |
| *H. filamentosum* | SU-1 | AM168100.1 | SSU |
| *H. oculare* |  | HQ141497.1 | SSU |
| *Polysphondylium fuscans* | Sweden-11D | JX173877.1 | SSU |
| *P. violaceum* | 209 | HQ141486.1 | SSU |
| *P. violaceum* | [WS-7](https://www.ncbi.nlm.nih.gov/nuccore/LC159233.1/) | LC159233.1 | ITS |
| *Raperostelium gracile* | TNS-C-183 | AM168078.1 | SSU |
| *R. monochasioides* | HAG653 | AM168052.1 | SSU |
| *R. ohioense* | Okla4C | HQ141493.1 | SSU |
| *R. potamoides* | FP1A | AM168069.1 | SSU |
| *R. tenue* | Pan52 | AM168076.1 | SSU |
| *R.* [*tenue*](https://www.ncbi.nlm.nih.gov/nuccore/AM168094.1/) | PJ6 | AM168094.1 | SSU |
| *Rostrostelium ellipticum* | AE2 | AM168112.1 | SSU |
| *Synstelium polycarpum* | VE1b | AM168057.1 | SSU |
| *S. polycarpum* | [OhioWILDS](https://www.ncbi.nlm.nih.gov/nuccore/AM168058.1/) | AM168058.1 | SSU |
| *Tieghemostelium lacteum* |  | AM168045.1 | SSU |
| *T. menorah* | M1 | AM168073.1 | SSU |

**Supplementary Table S3** Primers used to amplify the two genetic markers used in the present study.

| Primer name | 5’-3’ Primer sequence | Reference | PCR program |
| --- | --- | --- | --- |
| SSU |  |  | 95 C-5’, 25x(95 C-30’’, 56 C-1’, 72 C-2’), 72 C-10’ |
| 18SF-A | 5’AACCTGGTTGATCCTGCCAG3’ | Medlin *et al*. 1988^1^ |
| 18SR-B | 5’TGATCCTTCTGCAGGTTCAC3’ | Medlin *et al*. 1988^1^ |
| D542F | 5’ACAATTGGAGGGCAAGTCTG3’ | Schaap *et al*. 2006^2^ |
| D1340R | 5’TCGAGGTCTCGTCCGTTATC3’ | Schaap *et al*. 2006^2^ |
| ITS |  |  | 95 C-5’, 25x(94 C-1’, 50 C-1’, 72 C-2’), 72 C-10’ |
| ITS1 | 5’TCC GTA GGT GAA CCT GCG G3’ | White *et al*. 1990^3^  Romeralo *et al*. 2010^4^ |
| ITS4 | 5’TCC TCC GCT TAT TGA TAT GC 3’ | White *et al*. 1990^3^  Romeralo *et al*. 2010^4^ |


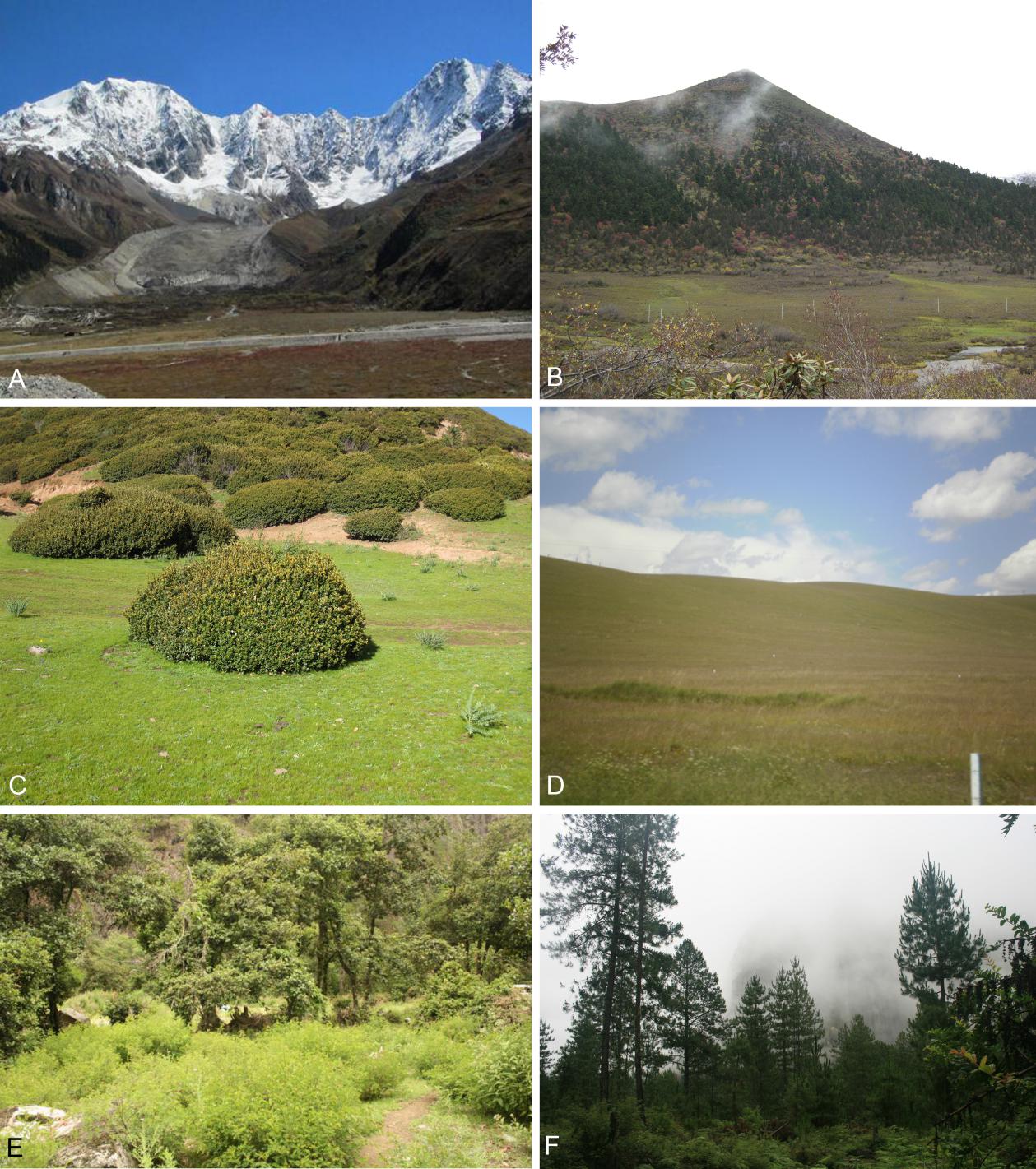


**Supplementary Figure S1** Different habitats on the Qinghai-Tibet Plateau. (A) Snowy mountains at high elevations; (B) mixed forest; (C) alpine grassland; (D) grassland; (E) broadleaf forest; (F) coniferous forest.
